# Supplementary material for: Complexome profiling of the Chlamydomonas psb28 mutant reveals TEF5 as an early PSII assembly factor
Source: Plant Cell. 2025 Mar 17;37(6):koaf055. doi: 10.1093/plcell/koaf055 (PMC12164590; doi:10.1093/plcell/koaf055)
Supplement: koaf055_Supplementary_Data [file koaf055_supplementary_data.zip › psb28_interactive.zip!/plots/intensity/qualityPlots/peptides/NTR3_Cre07.g355600.t1.1.html]

### Cre07.g355600.t1.1

Trivial name: NTR3  
SeqCoverage [median]: 15.90  
Mass: 36.53 kDa  
  
 

| Sequence | MissedCleavage | Mass [Da] | wtIntensity | muIntensity | Score |
| --- | --- | --- | --- | --- | --- |
| IYSETVDSIDTSR | 0 | 1484.70 | 751.48 | 1322.84 | 135.66 |
| NKPIAVIGGGDSAMEEATFLTK | 1 | 2248.14 | 880.91 | 0.00 | 57.29 |
| TADKEVTADSLIIATGAVAR | 1 | 2001.07 | 1510.76 | 3885.77 | 95.81 |
|  |  |  | 3143.1 | 5208.6 |  |
